# Supplementary material for: Cell-specific DNA methylation in human alpha and beta cells regulates gene expression in type 2 diabetes
Source: Nat Metab. 2026 Apr 24;8(4):957–80. doi: 10.1038/s42255-026-01498-9 (PMC13121032; doi:10.1038/s42255-026-01498-9)
Supplement: Supplementary file 1 — Supplementary Files 1–4. [file 42255_2026_1498_MOESM1_ESM.pdf]

nature metabolism

Supplementary information https://doi.org/10.1038/s42255-026-01498-9

Cell-specific DNA methylation in human alpha and beta cells regulates gene expression in type 2 diabetes

In the format provided by the authors and unedited

Supplementary File 1. Oxygen consumption rate measurements.

Mitochondrial oxygen consumption rates (OCR) were evaluated with an XFe24 extracellular flux analyzer (Agilent Technologies, Santa Clara, CA, USA). ~70,000 INS-1 β-cells were seeded on poly-D-lysine (1mg/ml) coated XF24 24-well culture microplates in 100 μL complete RPMI medium without antibiotics, followed by transfection with a siRNA targeting *Adcy9* or a negative control siRNA, or transduction with lentiviral vectors conferring expression of human *ONECUT2* or GFP as described in the main text. Next, cells were starved in SAB containing 2.8 mM glucose for 2 h and OCR was measured in unbuffered SAB (i.e., without bicarbonate and HEPES) every 3 min for 90 min. OCR was measured at basal glucose (2.8 mM glucose) and after addition of 16.7 mM glucose, 5 μM oligomycin, 4 μM Carbonyl cyanide ptrifluoromethoxyphenylhydrazone (FCCP), and 1 μM Rotenone/Antimycin A. The proportion of respiration driving ATP synthesis and proton leak were determined by addition of oligomycin. The uncoupler, FCCP, was added to determine maximal respiratory capacity. Rotenone and antimycin A were added to block the transfer of electrons from complex 1 to ubiquinone and through complex 3, respectively, allowing determination of non-mitochondrial oxygen consumption. Wave Seahorse Software and Seahorse Analytics online tool (seahorseanalytics.agilent.com) were used to analyze the data. Non-mitochondrial respiration was subtracted, and OCR data normalized to total protein content in the cells as measured by BCA assay.

Supplementary File 2. Gene expression microarray of *ADCY9*-deficient or *ONECUT2*-overexpressing human islets

Total RNA was extracted from human islets after silencing *ADCY9* using siADCY9 (N=5 donors), or lentiviral overexpression of *ONECUT2* (OE-*ONECUT2*, N=6 donors), by using the miRNeasy isolation kit (QIAGEN, Hilden, Germany) according to the manufacturer's recommendations. RNA quality was assessed by Agilent Technologies 2200 Tapestation and concentrations were measured by NanoDrop ND-1000 Spectrophotometer (ThermoFisher Scientific, Waltham, MA, USA). 100 ng of total RNA was used to generate amplified sense strand cDNA targets using GeneChip® WT Plus Reagent Kit (ThermoFisher Scientific) followed by fragmentation and labelling of 5.5 ug sscDNA. 2.3 μg of fragmented and labelled sscDNA target was hybridized to Clariom™ S Human/Rat Arrays for 16 h at 45°C under rotation in Affymetrix Gene Chip Hybridization Oven 645 (ThermoFisher Scientific), at

Bioinformatics and Expression analysis core facility (BEA), Karolinska Institute, Huddinge, Sweden. Washing and staining was carried out on Affymetrix GeneChip® Fluidics Station 450 (ThermoFisher Scientific), according to the manufacturer's protocol. The fluorescent intensities were determined with Affymetrix GeneChip Scanner 3000 7G (ThermoFisher Scientific). Data were processed using the Transcriptome Analysis Console (TAC) and the SST-RMA analysis method including log2-transformation, quantile normalization, background correction and summarization. Annotations are based on build hg38/GRCh38 and were collected from TAC. Student's t-tests (paired) were used to detect differentially expressed genes (DEGs) between control islets and islets treated with siADCY9 or OE-*ONECUT2*.

The expression microarray data from the *ADCY9*-deficient or *ONECUT2*-overexpressing islets were also analyzed by gene set enrichment analysis (GSEA) using R packages clusterProfiler (v.4.10.0) and enrichplot (v.1.22.0). Here, we used all probes on the array corresponding to transcripts with identifiers. To generate a gene list, probes with the same Entrez GeneID annotation were handled by keeping the probe where the mean expression value of the control was the highest. Then probes with multiple Entrez GeneID annotations were split, and again duplicates removed by keeping the max control expression value. The updated probes were then ranked according to the t-statistics in the paired t-test in descending order. The gseGO function from clusterProfiler was used for gene set enrichment with parameters OrgDb='org.Hs.eg.db', ont='BP', pvalueCutoff=0.05, pAdjustMethod='BH', by='fgsea', seed=TRUE, with set.seed(100) and org.Hs.eg.db (v.3.18.0). The function, pairwise_termsim from R package enrichplot was used to create a similarity matrix. The setReadable function with parameter OrgDb='org.Hs.eg.db' was used to obtain gene symbols for the Entrez_IDs of the tabular output.

The emapplot function from enrichplot was used to visualize the results as an enrichment map, with set.seed(100). For *ADCY9*, all gene sets were included, while for *ONECUT2*, a subselection of 23 gene sets was chosen to be displayed, using the parameter showCategory. Other parameters used for both emapplots were list(layout='grid'), color='NES, repel=TRUE, shadowtext=FALSE. For *ADCY9*, additionally cluster.params=list(cluster=TRUE), and for *ONECUT2*, group_category=TRUE, and nCluster=3.

The expression microarray data for OE-*ONECUT2* and the identified DEGs were used to generate co-expression networks using the R weighted correlation network analysis (WGCNA) package (v. 1.73). The analysis was performed using the blockwiseModules function with

parameters networkType = ‘unsigned’, maxBlockSize=30000, TOMType=’unsigned’, power=9, mergeCutHeight=0.25, minKMEtoStay=0, minModuleSize=20, deepSplit=2, pamRespectsDendro=F, numericLabels=TRUE, randomSeed=2345, verbose=3. Default settings were selected for the rest of the parameters. A soft-thresholding power of 9 was selected as recommended in the WGCNA package FAQ. A similarity matrix was constructed by calculating the correlation coefficient of each gene using Pearson’s correlation. Considering both positively and negatively correlated genes as connected (unsigned network type), a power function was used to transform the similarity network into an adjacency matrix. A topological overlap matrix was obtained from the adjacency matrix using the TOMsimilarityFromExpression function with parameters power=9, networkType=’unsigned’, TOMType=’unsigned’, using all genes in the same module as OE-*ONECUT2*, thus all 114 DEGs with q<0.1 were included. To generate node and edge files to a format suitable for importing to Cytoscape (v.3.10.3), the WGCNA function exportNetworkToCytoscape was run with the parameters weighted=TRUE and threshold=0.55, with the topological overlap matrix as input file. For visualization in Cytoscape, the obtained network was divided up into two circles, one with stronger connections to *ONECUT2* based on the threshold of 0.55, and one with connections that didn’t pass the threshold of 0.55 to *ONECUT2*. The latter were kept in the network due to strong connections to other genes within the module, in a second circle.

Supplementary File 3. Rescue experiment in human EndoC-βH1 β-cells exposed to high glucose plus palmitate.

One day before treatment and transfection, 180,000 EndoC-βH1 β-cells were seeded per well in a 48-well plate containing 150 μL DMEM medium without antibiotics (penicillin/streptomycin). The following day, cells received either fresh 150 μL control media or media containing high glucose plus palmitate (HGP, 19 mM glucose + 1 mM palmitate, mimicking diabetogenic conditions) with or without Silencer® Select Pre-Designed siRNA against *ONECUT2* (50 nM; s18176; ThermoFisher Scientific) using Silencer® Select Negative Control No. 2 siRNA as negative control (50 nM; 390846; ThermoFisher Scientific). Palmitate was administered conjugated to BSA (CC-29558), and unconjugated BSA (CC-29556, both Cayman Chemical, Ann Arbor, MI, USA) was used as control. A final transfection volume of 200 μL per well contained 50 nM siRNA in Opti-MEM reduced serum media and 0.5 μL Lipofectamine RNAiMAX. A second transfection was performed 24 h after the first

transfection. All functional experiments were performed 72 h after the first transfection, including measurement of insulin secretion. Confluent plates were carefully washed twice with 1 mL prewarmed SAB, pH 7.2 (1.16 mM MgSO4, 4.7 mM KCl, 1.2 mM KH2 PO4, 114 mM NaCl, 2.5 mM CalCl2, 25.5 mM NaHCO3, 20 mM HEPES, and 0.2% BSA), containing 1 mM glucose. Cells were then preincubated in 0.5 mL fresh SAB with 1 mM glucose for 2 h. The cells were stimulated in 0.25 mL SAB with 1 or 20 mM glucose at 37°C for 1 h . Although EndoC-βH1 β-cells are normally starved overnight before insulin secretion experiments to ensure a full response to glucose, we didn't starve the cells as this would interrupt the HGP treatment. Insulin was measured by ELISA (10-1113-01, Mercodia, Uppsala, Sweden), and secreted insulin was normalized to the total protein as determined by BCA Protein Assay kit (ThermoFisher Scientific). *ONECUT2* silencing was confirmed by qPCR as described in the main text.

Supplementary File 4. Analysis testing whether overlapped sets display statistically significant enrichment.

To show if overlapped sets display statistically significant enrichment, we used gene set enrichment hypergeometric tests for gene overlaps and region-based permutation tests for genomic region overlaps. Enrichment of T2D-associated genes found by GWAS within gene sets of interest (genes associated with DMRs/DEGs) was assessed with a one-tailed hypergeometric test by using R (v4.4, phyper) with the formula $P(X \geq k)$ = phyper(k - 1, m, n, K, lower.tail =FALSE), where m is the number of genes in the reference set, n is the number of genes not in the reference set (i.e., gene universe – m), K is the size of the query set, and k is the observed overlap. To define the background gene universe for each set of DMR-associated genes, we collected all single CpGs used to define the corresponding DMR set and annotated them to genes in the same way as the DMRs. In the case of overlaps with the found DEGs between α- versus β-cells, all the genes that were expressed in α- and β-cells (as defined in the section RNA Sequencing) were used as the background gene universe. Both methods ensured that the background represented the complete set of genes capable of being detected and assessed for differential methylation/expression, respectively. To quantify the magnitude of the association/overlap, an enrichment score (fold enrichment) was calculated as the ratio of the observed overlap to the expected overlap, where the expected overlap is the product of the query and reference set sizes divided by the total universe size. To assess the statistical

significance of the spatial association between the different sets of genomic regions of interest (DMRs, TF binding, and Hi-C sites), permutation testing was performed using the R package regioneR (v 1.38). First, an empirical null distribution was generated by performing a total of 1,000 overlap iterations between the query and the reference set. In each iteration, the positions of the query regions were randomized across the genome while preserving their size and chromosomal distribution (per.chromosome = TRUE). Randomization was restricted to valid genomic regions by excluding masked areas as defined in the genome version in the BSgenome.Hsapiens.UCSC.hg38.masked R package (v1.4.5, build hg38). A region was counted as overlapping if it shared at least 1 base pair with the reference set (count.once = TRUE). To assess statistical significance, the number of observed overlaps was compared to the overlaps of the distribution of the randomized regions using an empirical p-value. For 1,000 permutations, the lowest achievable p-value is 0.001. Therefore, to further determine the strength of the association, a Z-score, defined as the standardized deviation from the mean of the permutation distribution, is also provided as output by the same tool.
