# Supplementary material for: Cell-specific DNA methylation in human alpha and beta cells regulates gene expression in type 2 diabetes
Source: Nat Metab. 2026 Apr 24;8(4):957–80. doi: 10.1038/s42255-026-01498-9 (PMC13121032; doi:10.1038/s42255-026-01498-9)
Supplement: Supplementary file 2 — Reporting Summary [file 42255_2026_1498_MOESM2_ESM.pdf]

nature portfolio

Corresponding author(s): Charlotte Ling

Last updated by author(s): 24/02/2026

# Reporting Summary

Nature Portfolio wishes to improve the reproducibility of the work that we publish. This form provides structure for consistency and transparency in reporting. For further information on Nature Portfolio policies, see our Editorial Policies and the Editorial Policy Checklist.

## Statistics

For all statistical analyses, confirm that the following items are present in the figure legend, table legend, main text, or Methods section.

| n/a | Confirmed | |
|---|---|---|
| ☐ | ☒ | The exact sample size (*n*) for each experimental group/condition, given as a discrete number and unit of measurement |
| ☐ | ☒ | A statement on whether measurements were taken from distinct samples or whether the same sample was measured repeatedly |
| ☐ | ☒ | The statistical test(s) used AND whether they are one- or two-sided<br>*Only common tests should be described solely by name; describe more complex techniques in the Methods section.* |
| ☐ | ☒ | A description of all covariates tested |
| ☐ | ☒ | A description of any assumptions or corrections, such as tests of normality and adjustment for multiple comparisons |
| ☐ | ☒ | A full description of the statistical parameters including central tendency (e.g. means) or other basic estimates (e.g. regression coefficient) AND variation (e.g. standard deviation) or associated estimates of uncertainty (e.g. confidence intervals) |
| ☐ | ☒ | For null hypothesis testing, the test statistic (e.g. *F*, *t*, *r*) with confidence intervals, effect sizes, degrees of freedom and *P* value noted<br>*Give P values as exact values whenever suitable.* |
| ☒ | ☐ | For Bayesian analysis, information on the choice of priors and Markov chain Monte Carlo settings |
| ☐ | ☒ | For hierarchical and complex designs, identification of the appropriate level for tests and full reporting of outcomes |
| ☐ | ☒ | Estimates of effect sizes (e.g. Cohen's *d*, Pearson's *r*), indicating how they were calculated |

*Our web collection on statistics for biologists contains articles on many of the points above.*

## Software and code

Policy information about availability of computer code

| | |
|---|---|
| Data collection | Illumina NextSeq 500: NextSeq Control Software v. 4.0.1<br>NovaSeq 6000: NovaSeq Control Software v1.7.5 |

Data analysis

WGBS: Real Time Analysis v3.3, Sequencing Analysis Viewer v2.4.7, R (v4.1.3), bioconductor-annotatr (v1.20.0), bioconductor-txdb.hsapiens.ucsc.hg38.knowngene (v3.14.0), bioconductor-org.hs.eg.db (v3.14.0), bioconductor-bsseq (v1.30.0), bioconductor-dmrseq (v1.14.0), trim-galore (v0.6.6), fastqc (v0.11.9), multiqc (v1.12), cutadapt (v3.2), bowtie2 (v2.4.2), samtools (v1.12), bismark (v0.23.0), bamtools (v2.5.1)
EPIC arrays: R (v.4.6.0), Minfi (v.1.57.0), wateRmelon (v2.17.0)
RNA-seq: R (v4.2.2) with bioconductor-genomicfeatures (v1.50.22), bioconductor-tximport (v1.26.0), bioconductor-deseq2 (v1.38.0), bioconductor-genomeinfodb (v1.34.8), trim-galore (v0.6.7), cutadapt (v3.4), salmon (v1.5.2), fastqc (v0.11.9), multiqc (v1.11)

Overlap between DMRs and other data: HOMER (v.4.11, http://homer.ucsd.edu/homer/motif/), R (v.4.4.1),Bioconductor GenomicRanges (v1.54.1 and v.1.58.0), R package VennDiagram (v.1.7.3). annotatr (v.1.32.0), regioneR (v.1.38), BSgenome.Hsapiens.UCSC.hg38.masked (v.1.4.5), phyper (v.4.4)

Expression arrays: Transcriptome Analysis Console (v4.0), R packages clusterProfiler (v.4.10.0) and enrichplot (v.1.22.0), R WGCNA package (v. 1.73), Cytoscape (v.3.10.3)
Functional experiments: GraphPad Prism (v10)
The computer code used to generate the results described in the methods section under DNA methylation analysis and Statistical methods is deposited in Zenodo (https://doi.org/10.5281/zenodo.18681936). Other details are available from the corresponding author upon on request.

For manuscripts utilizing custom algorithms or software that are central to the research but not yet described in published literature, software must be made available to editors and reviewers. We strongly encourage code deposition in a community repository (e.g. GitHub). See the Nature Portfolio guidelines for submitting code & software for further information.

# Data

Policy information about availability of data

All manuscripts must include a data availability statement. This statement should provide the following information, where applicable:

- Accession codes, unique identifiers, or web links for publicly available datasets
- A description of any restrictions on data availability
- For clinical datasets or third party data, please ensure that the statement adheres to our policy

The WGBS and RNA-seq data generated from human α- and β-cells were deposited in Zenodo (https://doi.org/10.5281/zenodo.18656853) and the LUDC repository (https://www.ludc.lu.se/resources/repository, accession numbers: LUDC2025.08.1 for the α-cell WGBS data, LUDC2025.08.3 for the β-cell WGBS data, LUDC2025.08.4 for the α-cell RNA-seq data, and LUDC2025.08.5 for the β-cell RNA-seq data). Data can be requested through the repository portal and individual-level data from the human pancreatic islets are not publicly available due to ethical and legal restrictions related to the Swedish Biobanks in Medical Care Act, the Personal Data Act and European Union's General Data Protection Regulation and Data Protection Act. Source data are provided with this paper. The human α- and β-cell RNA-seq data used for validation is available in Gene Expression Omnibus GEO Accession viewer (GSE67543).

The gene expression microarray data from ADCY9-deficient or ONECUT2-overexpressing human islets are available in Gene Expression Omnibus GEO Accession viewer (accession numbers GSE319105 and GSE319109, respectively).

Data reproducibility: We have used several different methods/models to test the reproducibility of our data. For example, the global RNA-sequencing data was replicated via comparisons with published data (PMID 25931473 and 36656641), while the ONECUT2 overexpression in human beta-cells from people with T2D was replicated in islets from a rat model of diabetes. For the DNA methylation data in human alpha- and beta-cells, we could validate sex-associations based on our previous study in whole human islets (PMID 25517766), and age-associations based on our previous paper in whole islets (PMID 27029739), and T2D-associations based on our previous paper in whole human islets (PMID 38086799). Moreover, the global methylation patterns in the sorted alpha- and beta-cells cells are similar to the pattern in whole islets (PMID 28052964). For the functional analyses, we focus mainly on ADCY9 and ONECUT2. Manipulation of these two genes resulted in similar phenotypes regarding glucose-stimulated insulin secretion in both human islets and clonal beta-cells.

# Research involving human participants, their data, or biological material

Policy information about studies with human participants or human data. See also policy information about sex, gender (identity/presentation), and sexual orientation and race, ethnicity and racism.

Reporting on sex and gender

The findings of this project are based on inclusion of both sexes, determined by self-reporting and concordant genetic testing.
Sex is considered as a co-variate in genome-wide analyses of pre-T2D/T2D-associations in human alpha- and beta-cells.
The number of males and females in all analyses is presented in Supplementary Table 1. Briefly, the LUDC Islet Sorting Cohort consists of islets from 17 men and 7 women.

Reporting on race, ethnicity, or other socially relevant groupings

All pancreatic islets are from donors from The Nordic Network for Clinical Islet Transplantation Program (www.nordicislets.org) from Scandinavia, a defined geographic region. Clustering of existing GWAS data from this population suggests that a small number of donors are of non-Scandinavian descent. There is no reporting on any other socially relevant groupings.

Population characteristics

Regarding the LUDC Islet Sorting Cohort: 24 donors with an age of 64.0 (36-81) years, BMI of 25.8 (18.9-32.7) kg/m2, and HbA1c of 38.0 (22-49) mmol/mol.
The ethical permits do not allow sharing individual level clinical data in public domains.

Recruitment

Donors of pancreatic islets were from the Scandinavian Transplantation Unit and included multi-organ human donors. Islets were included in this study when not used for transplantation due to clinical reasons. There may be a bias in who is willing to transplant organs for research. However, it is impossible to dissect how such bias would impact our results.

Ethics oversight

All procedures regarding the human pancreatic islets were approved by the Swedish Ethical Review Authority (permit numbers 2007-05 and 2011-263). The studies followed the Helsinki Declaration and written informed consent was obtained from pancreatic islet donors or their relatives

Note that full information on the approval of the study protocol must also be provided in the manuscript.

# Field-specific reporting

Please select the one below that is the best fit for your research. If you are not sure, read the appropriate sections before making your selection.

☒ Life sciences ☐ Behavioural & social sciences ☐ Ecological, evolutionary & environmental sciences

For a reference copy of the document with all sections, see nature.com/documents/nr-reporting-summary-flat.pdf

# Life sciences study design

All studies must disclose on these points even when the disclosure is negative.

| | |
|---|---|
| Sample size | Sample size was based on our previous studies where we have analyzed DNA methylation and gene expression in human islets (PMID: 28052964), and performed gene knockdown and/or overexpression experiments (e.g. 36656641 and 38086799). |
| Data exclusions | WGBS data was excluded based on low coverage as this makes the data unreliable |
| Replication | The global RNA-sequencing data was replicated via comparisons with published data (PMID 25931473 and 36656641), while the ONECUT2 overexpression was also replicated in a rat model of diabetes.<br>For the generation of methylation data in whole human islets we have previously used several methods; EpiTYPER, WGBS, methylation arrays, and pyrosequencing. The data produced by the thee methods have always yielded data that correlate very strongly (see for example PMID 38086799 and 28052964). Moreover, the global methylation patterns in the sorted cells is very similar to the pattern in whole islets (PMID 28052964).<br>For the functional analyses, we focus mainly on ADCY9 and ONECUT2. Manipulation of these two genes resulted in similar phenotypes regarding glucose-stimulated insulin secretion in both human islets and clonal beta-cells. |
| Randomization | Islet samples were allocated into experimental groups (control, pre-T2D, or T2D) by the donor having a T2D diagnosis or not, or by their HbA1c.<br>Samples were allocated to sex based on self-reporting and concordant genetic testing. |
| Blinding | During the genome-wide DNA methylation and gene expression experiments the samples were anonymous to the technicians who ran the analyses and hence they did not know which group samples belonged to. However, during for example qPCR and Western blot analysis, the technician needed to know the order the samples were loaded on plates and gels and then blinding was not possible. |

# Reporting for specific materials, systems and methods

We require information from authors about some types of materials, experimental systems and methods used in many studies. Here, indicate whether each material, system or method listed is relevant to your study. If you are not sure if a list item applies to your research, read the appropriate section before selecting a response.

## Materials & experimental systems

| n/a | Involved in the study | |
|---|---|---|
| ☐ | ☒ | Antibodies |
| ☐ | ☒ | Eukaryotic cell lines |
| ☒ | ☐ | Palaeontology and archaeology |
| ☐ | ☒ | Animals and other organisms |
| ☐ | ☒ | Clinical data |
| ☒ | ☐ | Dual use research of concern |
| ☒ | ☐ | Plants |

## Methods

| n/a | Involved in the study | |
|---|---|---|
| ☒ | ☐ | ChIP-seq |
| ☐ | ☒ | Flow cytometry |
| ☒ | ☐ | MRI-based neuroimaging |

## Antibodies

Antibodies used

Antibodies used for FACS: guinea pig anti-insulin (A0564, Lot 10119708, 1:600 dilution, Dako), mouse anti-glucagon (MAB1249, Lot HSQ0416111, clone number 181402, 1:200 dilution, R&D Systems), donkey anti-guinea pig Alexa-647 (706-605-148, Lot 129003, 1:400 dilution, Jackson Immunoresearch) and donkey anti-mouse Alexa-488 (A-21202, Lot 1644644, 1:400 dilution, Invitrogen).

Antibodies used for western blot: Anti-6X His tag (#ab213204, lot 1000344-30, clone number EPR20547, 1:500 dilution, Abcam), anti-ADCY9/AC9 (#ab191423, lot GR269903-5, clone number EPR16188, 1:500 dilution, Abcam), total OXPHOS rodent Ab cocktail (#ab110413-MS604, lot 2101021674, 1:250 dilution, Abcam), horseradish peroxidase (HRP)-conjugated goat anti-rabbit (#7074, lot 32, 1:10,000 dilution, Cell Signaling Technology), and goat anti-mouse (#1706516, 719 1:2,000 dilution, Bio-Rad).

| | |
|---|---|
| Validation | All antibodies are commercially available and have been characterized by the manufacturers (and in several publications) for their reactivity in the appropriate species and for their compatibility to be used with the respective application. Conditions for blocking and antibody dilutions are stated in the method section. siRNA-silenced samples were used for validation of the anti-ADCY9 antibody (Supplementary Fig. 6c). For the overexpression experiments, we used GFP-transfected cells as validation (Supplementary Fig. 6d). For Western blot we also used molecular weight markers to identify the band(s) that migrated at the expected size of each respective protein analyzed. Validation details are also available on the manufacturers' websites. |

## Eukaryotic cell lines

Policy information about cell lines and Sex and Gender in Research

| | |
|---|---|
| Cell line source(s) | We used the rat INS-1 832/13 β-cell developed by Hohmeier, H.E. et al (PMID: 10868964) and the human EndoC-βH1 developed by Ravassard P. et al (PMID 21865645). INS-1 832/13 β-cells were developed from a male rat and EndoC-βH1 from a female fetus. |
| Authentication | It was verified that these cells are β-cells based on their ability to secrete insulin and respond to glucose with increased glucose-stimulated insulin secretion (only β-cells produce and secrete insulin). Additionally, the EPIC v.2.0 data from EndoC-βH1 also support β-cell origin as the degree of INS DNA methylation was low while GCG DNA methylation was high in promoter regions in cells not exposed to epigenetic editing. The cell lines were not authenticated using any additional authentication techniques. |
| Mycoplasma contamination | Cells were tested regularly for mycoplasma and were never contaminated. |
| Commonly misidentified lines (See ICLAC register) | No commonly misidentified cell lines were used in the study. |

## Animals and other research organisms

Policy information about studies involving animals; ARRIVE guidelines recommended for reporting animal research, and Sex and Gender in Research

| | |
|---|---|
| Laboratory animals | We used male GK rats, developed by selective breeding of Wistar rats (PMID: 32180184) and control Wistar rats from Janvier labs in France. Animals were kept in standard controlled housing conditions; 21–22°C, 55–65% humidity and 12-h light/12-h dark cycle and given standard chow (SAFE® A40, SAFE, Rosenberg, Germany) and water ad libitum. The animals were used at 11-13 weeks of age. |
| Wild animals | No wild animals were used in the study. |
| Reporting on sex | Regarding animals, we only use one sex. The main reason is to reduce the number of animals included in the experiments according to 3R principle (Replacement, Reduction and Refinement). It is previously known that both male and female GK rats have reduced insulin secretion. Moreover, in all our human experiments both sexes were included. |
| Field-collected samples | No field collected samples were used in the study. |
| Ethics oversight | Animal experiments were performed with permission of the Animal Ethics Committee of Lund University (Permit number 5.8.18-04115/2021) in accordance with the legal requirements of the European Community (86/609/EEC). |

Note that full information on the approval of the study protocol must also be provided in the manuscript.

## Clinical data

Policy information about clinical studies

All manuscripts should comply with the ICMJE guidelines for publication of clinical research and a completed CONSORT checklist must be included with all submissions.

| | |
|---|---|
| Clinical trial registration | not relevant for this study |
| Study protocol | not relevant for this study |
| Data collection | not relevant for this study |
| Outcomes | not relevant for this study |

## Plants

Seed stocks

not relevant for this study

Novel plant genotypes

not relevant for this study

Authentication

not relevant for this study

## Flow Cytometry

### Plots

Confirm that:

☒ The axis labels state the marker and fluorochrome used (e.g. CD4-FITC).

☒ The axis scales are clearly visible. Include numbers along axes only for bottom left plot of group (a 'group' is an analysis of identical markers).

☒ All plots are contour plots with outliers or pseudocolor plots.

☒ A numerical value for number of cells or percentage (with statistics) is provided.

### Methodology

Sample preparation

Samples were prepared according to the protocol in PMID:24594682, with minor changes:
Islets were centrifuge at 150 rcf(g) for 2 min at RT before being washed 3 times with PBS. Islet were then dispersed by addition of 5ml of TrypLE (#12604013, ThermoFisher Scientific) and incubattion in a water bath at 37° for 6-8 min (until the solution turns into a milky suspension). Cells were pipetted up and down a few times to break up cell clumps and complete media was then added. Cells were allowed to rest for 5 minutes before centrifugation (300g for 10min). Cells were then resuspended in media and passed through a 40µm cell strainer before being washed twice in PBC (300g for 5min).
Work surfaces, centrifuge, micropipettors, pipet-aid, and gloves were cleaned with RNaseZap. Cells were resuspended in fixation buffer (4% PFA, 0.1% saponin, and 2%RNase inhibitor, in PBS) and incubated under gentle agitation for 30min at 4 degrees. Cells were then centrifuged 3min at 3000g at 4°. before being washed twice in wash buffer (0.2% BSA, 0.1% saponin, 1% RNase inhibitor, in PBS). After the second wash, cells were resuspended in staining solution (1% BSA, 0.1% saponin, 5% RNase inhibitor, in PBS) with guinea pig anti-insulin (Dako, A0564, 1:600) and mouse anti-glucagon (R&D Systems, MAB1249, 1:200) and put on rotator at 4 degrees for 30 minutes. Cells were then washed twice in wash buffer before resuspension in staining buffer with donkey anti-guinea pig Alexa-647 (Jackson Immunoresearch, 706-605-148, 1:400) and donkey anti-mouse Alexa-488 (Invitrogen, 1:400). Tubes were wrapped in aluminum foil and put on rotator at 4 degrees for 30 minutes. Cells were then washed twice in wash buffer and resuspended in sorting buffer (0.5% BSA and 5% RNase inhibitor in PBS) and straining into a FACS tube with strainer cap before sorting.

Instrument

Aria Fusion (BD Biosciences, San Jose, CA, USA)

Software

FlowJo

Cell population abundance

We sorted 322,627±73,055 α-cells (purity 92.0±7.6%) and 311,550±61,250 β-cells (purity 96.5%±4.0) from islet preparations of 24 donors with or without T2D (cell number and purity from FloJo). The purity of the samples was confirmed by methylation and expression of GCG and INS, the main markers of α- and β-cells, respectively, in the WGBS and RNA-seq data.

Gating strategy

The gating strategy is displayed in Supplementary Fig 1a.

☒ Tick this box to confirm that a figure exemplifying the gating strategy is provided in the Supplementary Information.
