# Supplementary material for: Cell-specific DNA methylation in human alpha and beta cells regulates gene expression in type 2 diabetes
Source: Nat Metab. 2026 Apr 24;8(4):957–80. doi: 10.1038/s42255-026-01498-9 (PMC13121032; doi:10.1038/s42255-026-01498-9)

Extended Fig.6c

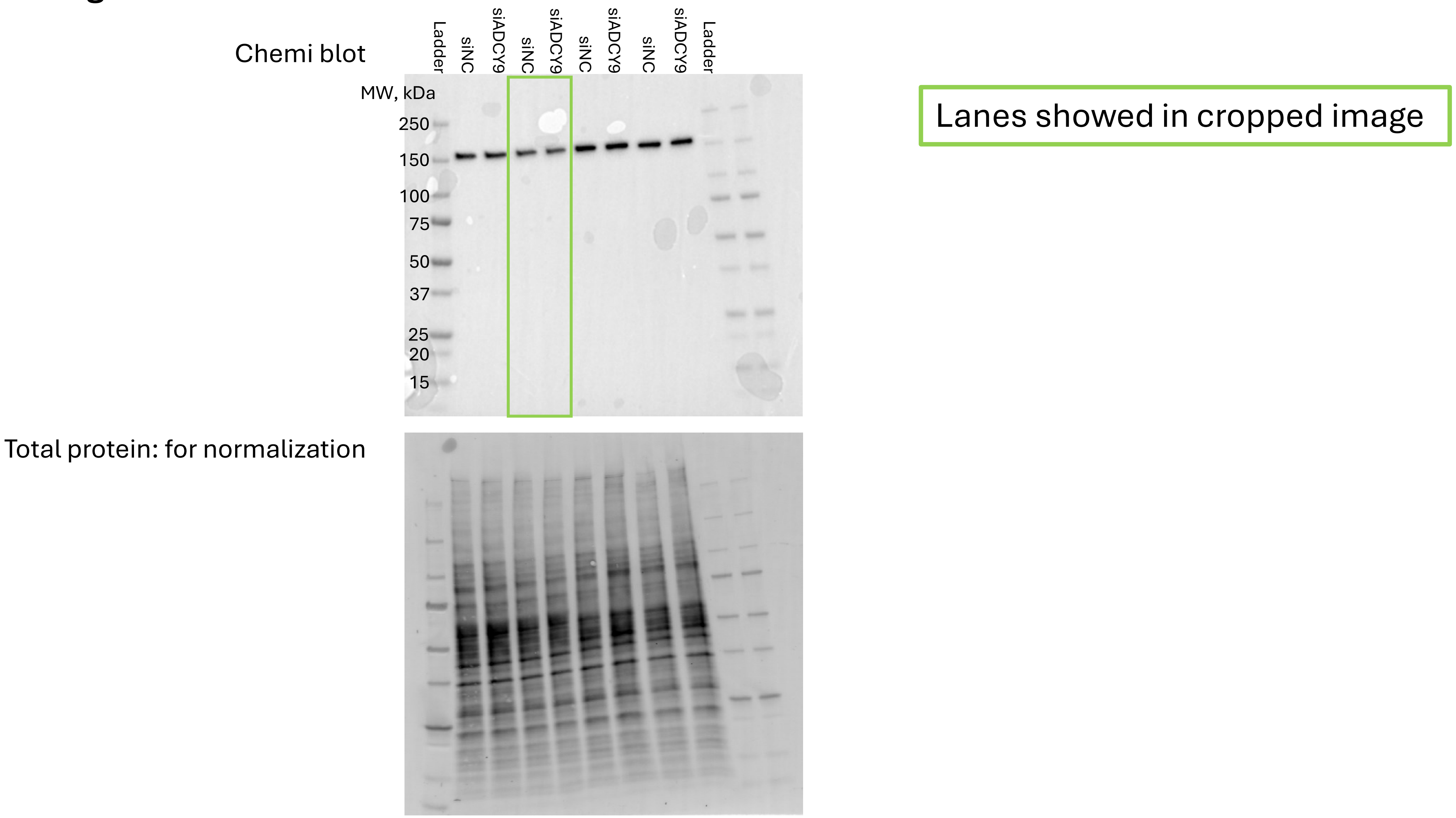

Extended Fig.6d

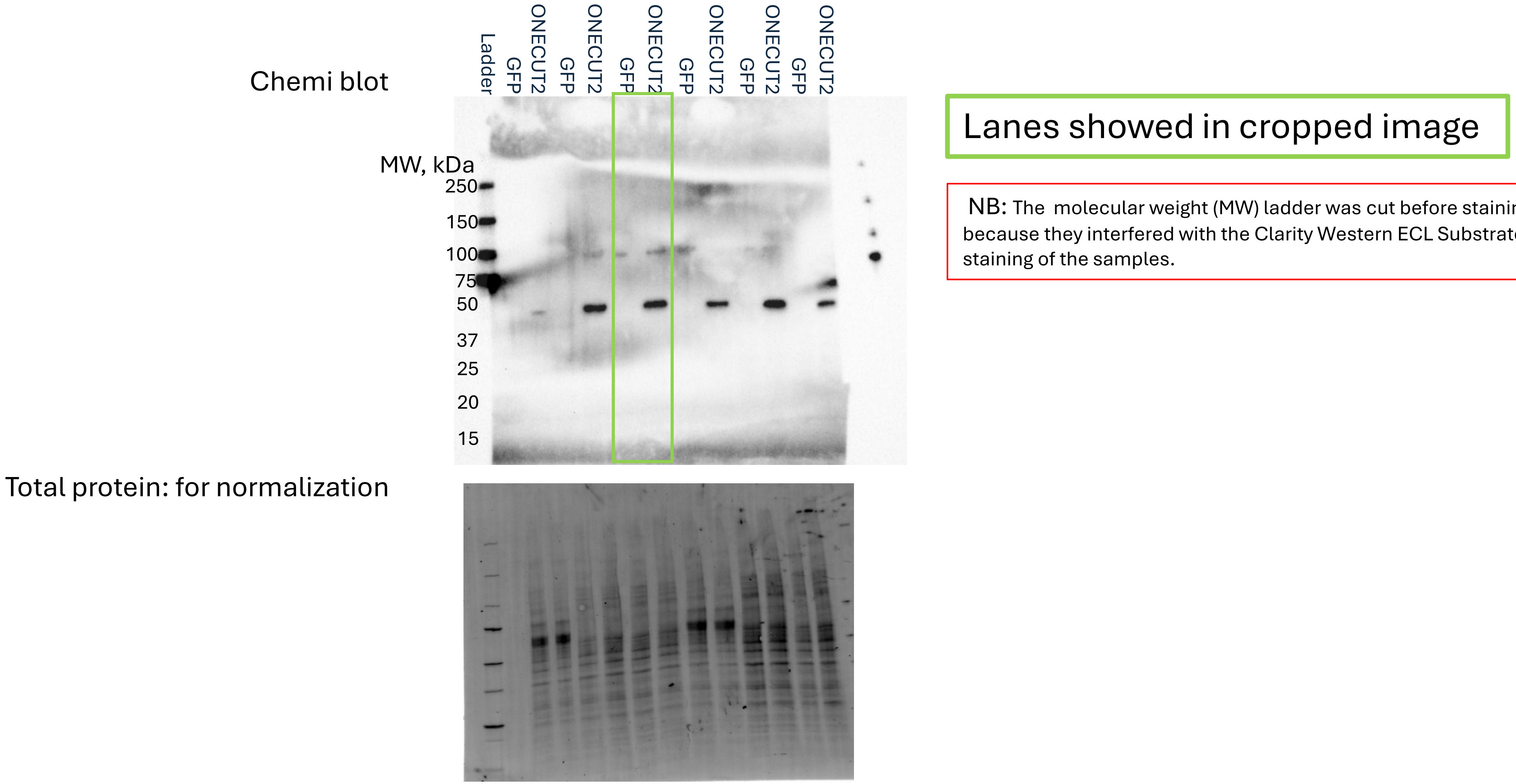

NB: The molecular weight (MW) ladder was cut before staining because they interfered with the Clarity Western ECL Substrate staining of the samples.

Extended Fig.6f

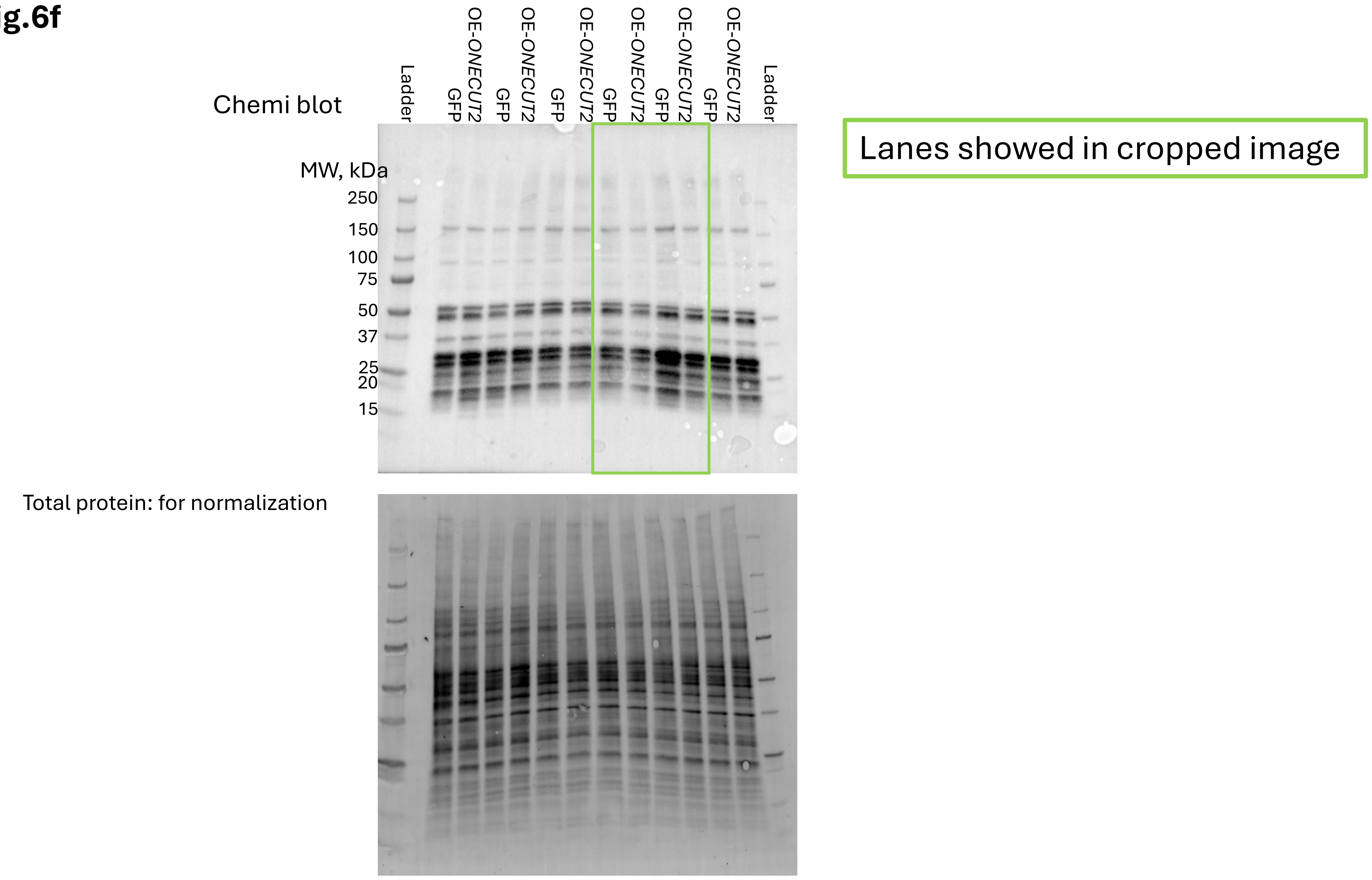

Supplement: Supplementary file 28 — Unprocessed western blots. [file 42255_2026_1498_MOESM28_ESM.pdf]
